# Transthoracic M-mode echocardiographic assessment of pulmonary vein-to-pulmonary artery ratio in healthy horses

**DOI:** 10.1371/journal.pone.0221154

**Published:** 2019-08-14

**Authors:** Domenico Caivano, Andrea Corda, Mark Rishniw, Maria Elena Giorgi, Maria Luisa Pinna Parpaglia, Maria Beatrice Conti, Francesco Porciello, Francesco Birettoni

**Affiliations:** 1 Department of Veterinary Medicine, University of Perugia, Perugia, Italy; 2 Department of Veterinary Medicine, University of Sassari, Sassari, Italy; 3 Veterinary Information Network, Davis, California, United States of America; 4 Department of Clinical Sciences, Cornell University, Ithaca, New York, United States of America; Universita degli Studi di Napoli Federico II, ITALY

## Abstract

Equine cardiovascular structures and function are routinely assessed by transthoracic echocardiography. Recently, investigators have described the echocardiographic visualization of equine pulmonary vein ostia. In companion animals, the right pulmonary vein (RPV) to right pulmonary artery (RPA) ratio has been used as an index to estimate the severity of cardiac diseases resulting in left ventricular volume overload. We sought to assess the feasibility of measuring RPV and RPA dimensions, and sought to provide various previously examined RPV and RPA variables in clinically healthy horses that could be used to assess cardiopulmonary disease status. Echocardiographic examination was prospectively performed in 70 healthy horses. The RPV and RPA were visualized using a modified right parasternal long-axis view and maximum and minimum diameters of both vessels were measured from 2D guided M-mode traces. The aortic diameter (Ao) was measured from the right parasternal short-axis view in early diastole. These measurements were then used to produce various ratio indices. RPV and RPA were imaged in all 70 horses. Median of the minimum and maximum RPV/RPA was 0.51 and 0.60, respectively. Median fractional dimensional change of vessels was 33% for RPV and 22% for RPA. The medians of the minimum and maximum RPV/Ao and RPA/Ao were 0.18, 0.28, 0.35 and 0.46, respectively. No relationships between either bodyweight or heart rate and any of the vein or artery variables were identified (maximum r^2^ = 0.04). Inter- and intra-observer measurement variability was very good for all RPV and RPA measurements. Measuring of RPV and RPA diameters using M-mode transthoracic echocardiography is feasible in healthy horses. Further studies of these variables in horses with cardiac diseases are needed to determine the clinical applicability and utility.

## Introduction

Equine cardiovascular structures and function are routinely assessed by transthoracic echocardiography [1,2]; left atrial (LA) and left ventricular diameters can provide useful information in horses with atrial fibrillation, aortic insufficiency and mitral regurgitation [1–3].

Recently, investigators have described the echocardiographic visualization of equine pulmonary vein ostia [4,5]. These authors suggested that the size and flow patterns of the pulmonary veins might help the assessment of cardiovascular disease in horses [4,5].

Investigators have evaluated the right pulmonary vein ostium (RPV) and right pulmonary artery ostium (RPA) in dogs and cats as an echocardiographic index for estimating severity of canine myxomatous mitral valve disease [6,7] and feline cardiomyopathies [8], and for identifying pulmonary hypertension in dogs [9,10]. These studies demonstrated that the RPV/RPA ratio, obtained from a modified right parasternal long axis view, imperfectly discriminated dogs with congestive heart failure from dogs with subclinical mitral valve disease [7,11]. However, selected RPV and RPA variables performed better than LA to aorta ratio in discriminating cardiomyopathic cats with and without congestive heart failure [8]. Whether similar vascular ratios would provide additional information in equine cardiac disease remains unknown.

However, to the authors’ knowledge, no studies exist describing echocardiographic assessment of the third pulmonary venous ostial and RPA diameters in healthy horses. Therefore, we sought to assess the feasibility of measuring the third pulmonary venous ostial and RPA dimensions, and to characterize previously reported pulmonary venous ostial and RPA variables in clinically healthy horses that could allow clinicians to assess cardiopulmonary disease status.

## Materials and methods

### Study population

We prospectively recruited 70 client-owned healthy horses from various farms in Italy (Sardinia, Umbria and Tuscany regions). Horses were considered healthy based on history, physical examination, routine electrocardiography and echocardiography. The study protocol was approved by the Ethics Committee of the University of Sassari (approval number 5731). Owners gave informed consent for their animals’ inclusion in the study.

### Echocardiography

**Fig 1:**
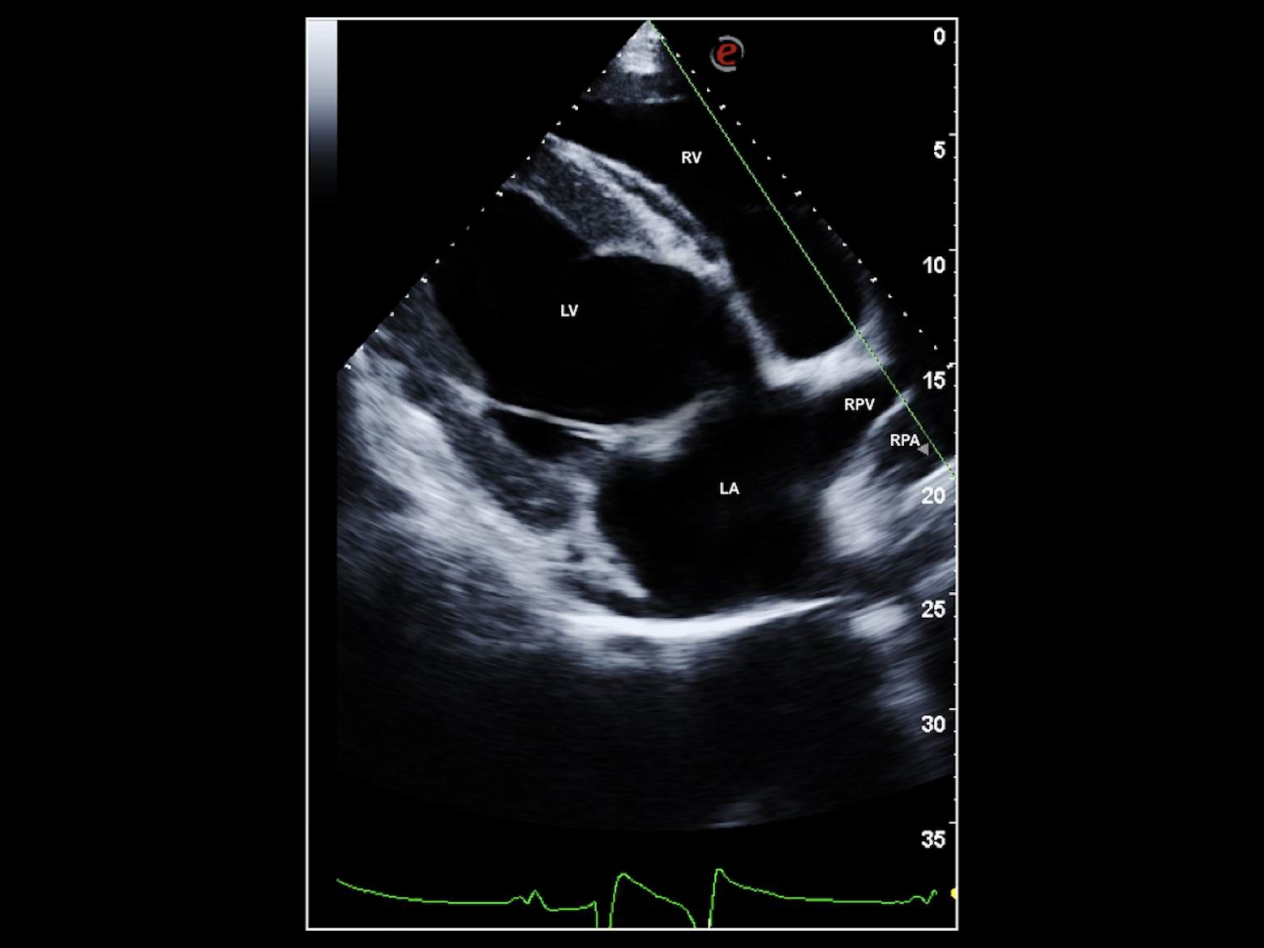
Echocardiographic image. Two-dimensional echocardiographic right parasternal long-axis view optimized for the third pulmonary vein ostium (RPV) and right pulmonary artery ostium (RPA) in a healthy horse, showing placement of the M-mode cursor (green line) for M-mode acquisition. RPV: right pulmonary vein ostium; RPA: right pulmonary artery ostium; RV: right ventricle; LA: left atrium; LV: left ventricle.

Echocardiograms were performed by 2 experienced sonographers (AC and MEG) using 2 different portable ultrasound units (MyLab 30 Gold and MyLab Alpha, Esaote, Italy) both equipped with multi-frequency 1–4 MHz phased array transducers. Echocardiographic examination was performed in all horses without sedation and in according to previously described imaging standards [12–14] with some modifications. Briefly, the sonographers modified the right parasternal long axis view as follows: to visualize the third pulmonary venous ostium and RPA, the sonographers moved the ultrasound probe slightly apically and angled it dorso-cranially, to obtain a somewhat oblique view of the heart base (Fig 1). To conserve nomenclature that has been adopted in companion animals, we refer to the third pulmonary venous ostium as the right pulmonary vein (RPV) throughout the remainder of this manuscript, as this ostium receives blood from the largest part of the right lung [4,5], and is analogous to the right pulmonary vein in dogs and cats [6–8].

**Fig 2:**
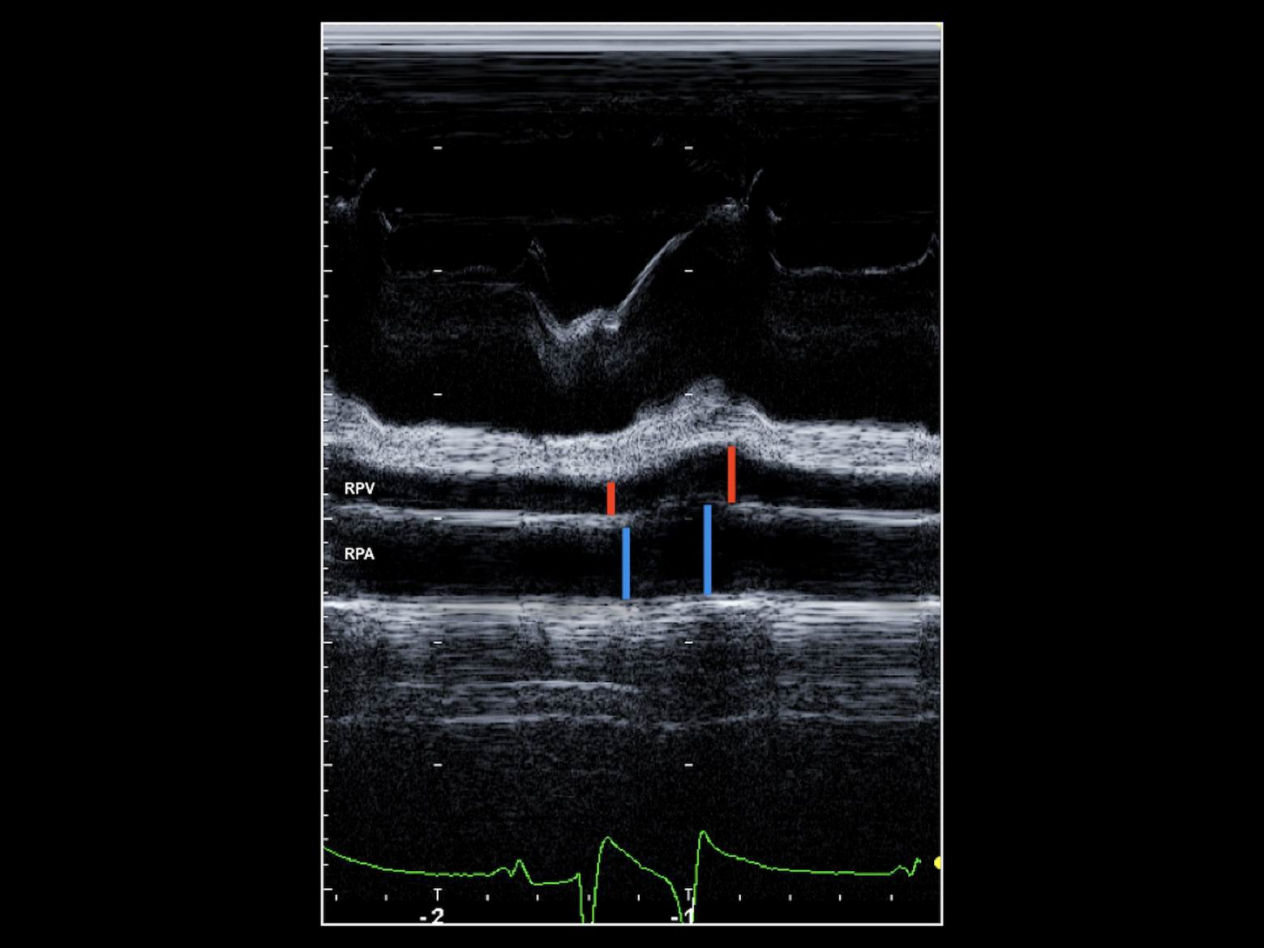
Echocardiographic image. M-mode echocardiographic image of the third pulmonary vein ostium (RPV) and right pulmonary artery ostium (RPA) obtained from the right parasternal long axis view. Red and blue lines show visually identified maximal and minimal diameters of the RPV and RPA, respectively, using the blood tissue interface method. RPV: right pulmonary vein ostium; RPA: right pulmonary artery ostium.

In this view, the RPA appeared as a circle just below the linear RPV. The sonographers then positioned the M-mode cursor for M-mode acquisition so that it perpendicularly bisected the RPV, where the venous walls were reasonably parallel, and to pass through the center of the adjacent RPA (Fig 1). The sonographers then acquired 2D guided M-mode traces that displayed at least two consecutive cardiac cycles and stored all data for subsequent off-line analysis (Fig 2).

**Fig 3:**
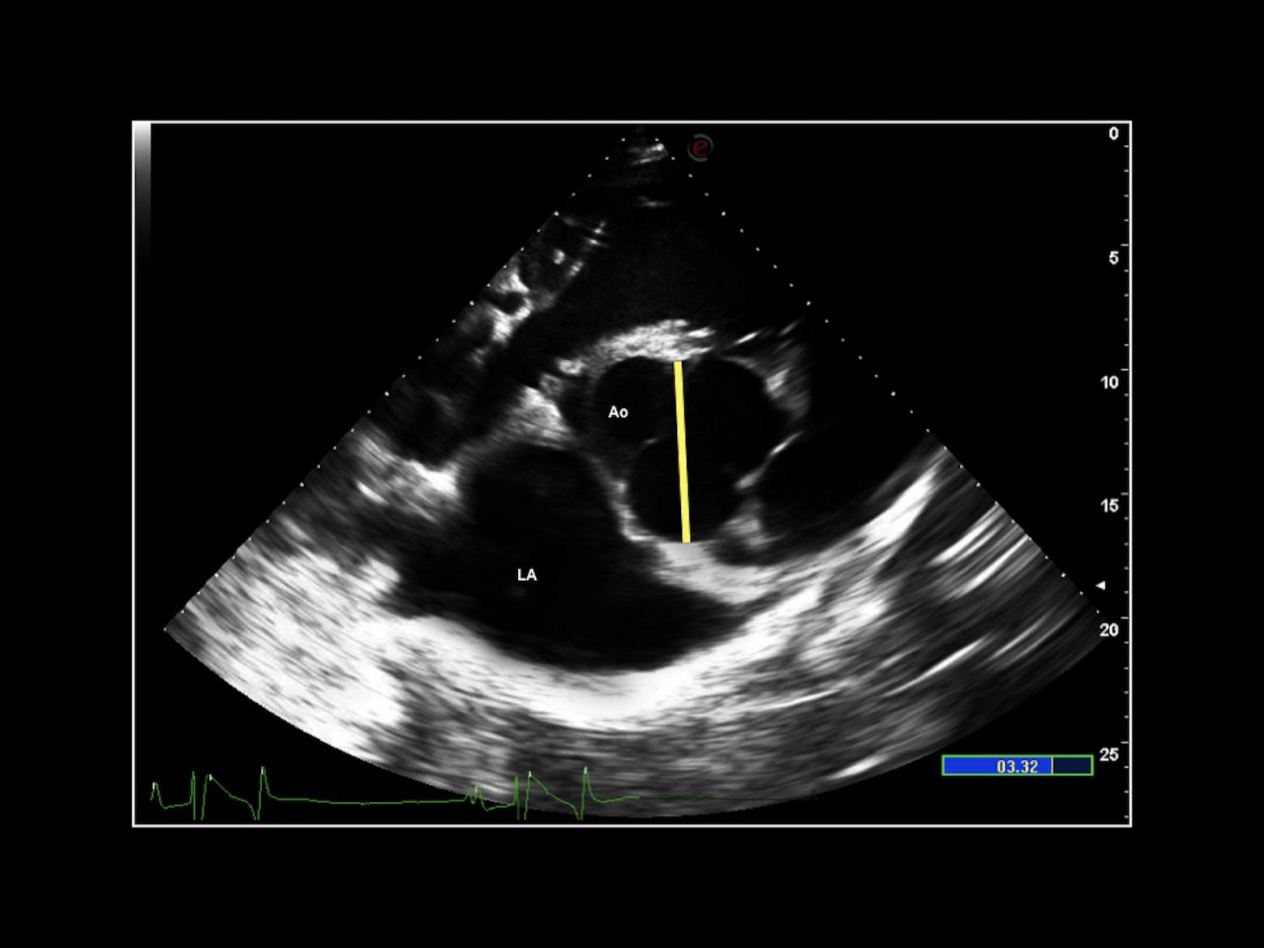
Echocardiographic image. Two-dimensional echocardiographic right parasternal short-axis view at the heart base. Aortic diameter (yellow line) was measured at aortic valve level, where the valve cusps were visible at the onset of the diastole. LA: left atrium; Ao: aorta.

Aortic diameter (Ao) was measured from the right parasternal short-axis view at aortic valve level, where the valve cusps were visible at the onset of the diastole (Fig 3), as previously described for dogs and cats [6,8].

Because no all horses tolerated electrocardiographic clips or lead wires, no simultaneous ECG tracing was recorded in all horses. At least 3 nonconsecutive cardiac cycles were stored in cine-loop format for each measured variable (RPV, RPA, Ao).

Because clinicians often perform echocardiography on horses “in the field” without simultaneous ECG monitoring, we opted to examine the RPV and RPA variables using mechanically timed events. One investigator (DC) measured all the echocardiographic variables off-line from archived M-mode images, at the subjective maximum (RPV_max_ and RPA_max_) and minimum diameters (RPV_min_ and RPA_min_) of both vessels, using the inner edge-to inner edge method, as previously described [6]. Three representative, nonconsecutive cardiac cycles were measured, and individual measurements were subsequently averaged. Cycles during marked transient tachycardia, following a sinus pause or a 2nd-degree atrioventricular block were excluded from analysis.

From the measured vessels diameters, we calculated the following variables:

- RPV_min_/RPA_min_: the ratio between RPV and RPA at their minimum diameter
- RPV_max_/RPA_max_: the ratio between RPV and RPA at their maximum diameter
- RPV_min_/Ao: the ratio between RPV at its minimum diameter and Ao
- RPV_max_/Ao: the ratio between RPV at its maximum diameter and Ao
- RPA_min_/Ao: the ratio between RPA at its minimum diameter and Ao
- RPA_max_/Ao: the ratio between RPA at its maximum diameter and Ao
- ΔRPV (fractional dimensional change - ΔRPV%): (RPV_max_-RPV_min_)/RPV_max_ × 100
- ΔRPA (fractional dimensional change - ΔRPA%): (RPA_max_-RPA_min_)/RPA_max_ × 100

### Data analysis

We assessed the distributions of the data on variables with a Shapiro-Wilk test. Because several of the variables did not conform to requirements for parametric reference intervals, we adopted a non-parametric approach for all variables to maintain consistency and avoid confusion [15] and presented the measures of central tendency as medians and interquartile ranges. To examine the effects of bodyweight (BW) and heart rate (HR) on the dependent variables, we drew scatter plots to visually examine the data. After examining for major violations of assumptions (and confirming that none existed), we performed linear regression analysis to determine if any substantive relationship existed.

To examine inter- and intra-observer variability in measuring the variables, ten studies were selected randomly (using a random number generator). Each investigator (AC and DC) then measured the minimal and maximal RPV and RPA dimension in each of these selected studies. One investigator (DC) measured the same studies on 2 separate occasions, separated by at least one month, and without knowledge of the previous measurements. Coefficients of variation are problematic when calculated for pairs of measurements, because the standard deviation of a pair of observations cannot be accurately determined. Therefore, we calculated the percent difference and the absolute difference between the 2 sonographers for each variable in each study and for the 2 measurements by the same sonographer as has been previously described [8,11].

## Results

The study population consisted in 27/70, female horses, 34/70 geldings and 9/70 stallions of the following breeds: Anglo-Arabian (n = 22), warmblood (n = 23), Arabian (n = 10), Quarter Horse (n = 6), Thoroughbred (n = 3), Italian Trotter (n = 2), Haflinger (n = 1), Holstein (n = 1), Appaloosa (n = 1) and Quarab (n = 1), with a median BW of 480 kg (range 300–670 kg) and a median age of 8 years (range 2–27 years). Body condition score ranged from 5 to 6 on 9-point scale (Henneke scale) in all horses. During the echocardiography, horses had a mean HR of 40±8 beats per minute. We were able to image and measure the RPV and RPA in all 70 horses. The M-mode tracing was characterized by multiple phasic deflections both of the RPV and RPA wall (Fig 2). Twenty out of 70 (28.6%) horses did not tolerate electrocardiographic clips or lead wires, so we could not acquire simultaneous ECG tracings. In the 50/70 (71.4%) horses with available ECG tracings, a positive RPV deflection began immediately after the QRS complex, reaching its azimuth near to the end of the T wave and then declined to a nadir just after the QRS complex (before the onset of the positive deflection). We additionally identified a positive deflection, followed by a gradual decrement in the RPA tracings. The positive deflection reached its azimuth during the T wave and declined to a nadir at the end of the QRS complex (Fig 2). In horses without ECG tracings, RPV_min_ and RPV_max_ were measured at the onset and at the azimuth of positive deflection, respectively. Similarly, RPA_min_ and RPA_max_ were measured at the onset and at the azimuth of positive deflection, respectively.

The medians and reference limits of the various RPV and RPA variables are displayed in Table 1.

**Table 1:** Reference intervals for right pulmonary vein and pulmonary artery variables in 70 clinically healthy horses.

Variable	Median	Lower reference limit (90% CI^a^)	Upper reference limit (90%CI)
RPV^b^_min_/RPA^c^_min_	0.51	0.32 (0.29–0.36)	0.81 (0.75–0.83)
RPV_max_/RPA_max_	0.60	0.41 (0.38–0.45)	0.87 (0.83–0.88)
RPV_min_/Ao^d^	0.18	0.11 (0.10–0.13)	0.27 (0.25–0.29)
RPV_max_/Ao	0.28	0.18 (0.17–0.19)	0.39 (0.37–0.40)
RPA_min_/Ao	0.35	0.26 (0.25–0.27)	0.46 (0.42–0.50)
RPA_max_/Ao	0.46	0.36 (0.34–0.37)	0.57 (0.53–0.59)
ΔRPV^e^ (%)	33	17 (17–19)	51 (48–53)
ΔRPA^f^ (%)	22	11 (10–12)	37 (34–39)

^a^CI–confidence interval

^b^RPV– third pulmonary vein ostium

^c^RPA–right pulmonary artery

^d^Ao –aorta

^e^ΔRPV–pulmonary vein fractional dimensional change

^f^ΔRPA–pulmonary artery fractional dimensional change

We identified no relationships between either BW or HR and any of the RPV or RPA variables (maximum r^2^ = 0.04) (S1 File).

Inter-observer measurement variability was very good for all RPV and RPA measurements. The largest percentage difference between the 2 sonographers was 8% (for RPV_min_). In all other measurements, the percentage difference was <7%. Similarly, intra-observer variability was very good. As with inter-observer variability, the RPV_min_ measurement had the greatest intra-observer variability, with the percentage difference approaching 8%, whereas all other measurements had a percentage difference <6% for intra-observer variability. When assessed on an absolute difference, the average measurements for RPV and RPA differed between sonographers and within sonographer by <1.5 mm.

## Discussion

We have, for the first time, demonstrated the feasibility of measuring RPV and RPA diameter in healthy horses and have also generated reference intervals for various RPV and RPA variables, as has previously been reported in dogs and cats. We could reliably image the RPV and RPA diameters by transthoracic echocardiography in healthy horses using a modified right parasternal long axis view to obtain the relevant M-mode images. We could easily detect the anatomical landmarks that corresponded to vessels identified by previous investigators [6,16]. Whether these variables will prove useful in assessing equine cardiac pathology, identifying congestive heart failure, or predicting success of cardioversion in atrial fibrillation, remains to be determined.

Because clinicians often perform echocardiographic evaluations of horses without simultaneous ECG monitoring, we opted to use mechanically timed events to measure RPV and RPA variables (minimal and maximal vessel distensions), rather than ECG-timed events. In horses with ECG available, minimum and maximum diameter of the RPV and RPA occurred before the onset and during the positive deflection of both vessels, respectively. Although mechanically timed events are subject to a “best guess” by the sonographer, our inter- and intra-observer variability analyses suggest that, at least in this study, both observers consistently identified maximal and minimal diameters of both vessels. Furthermore, previous investigators have demonstrated that mechanically or electrically timed RPV and RPA measurements work equally well in dogs and cats, and that the mechanically based method more accurately determined vessel fractional dimensional change [6,8].

We visualized phasic deflections of the RPV and RPA on the M-mode tracings of horses that resembled the M-mode deflections observed in dogs and cats [6,8]. Right pulmonary vein ostium diameter in the horse changes during the cardiac cycle, reflecting the different phases of LA function (reservoir, conduit and booster pump phase), as previously described in humans and small animals [6,8,17,18]. The RPV reaches its maximum diameter at ventricular end systole. Indeed, during ventricular systole, the mitral annulus is pulled apically and the LA acts as an aspiration pump. Thus, the LA, including the RPV, relaxes and is stretched apically with increasing of both volumes just prior to mitral valve opening [18]. The RPV diameter then decreases when the mitral valve opens, during the rapid ventricular filling phase. The RPV reaches its minimum diameter just before the ventricular systole: in humans and horses, atrial myocardial fibers, oriented circumferentially, extend into the pulmonary vein ostia [17,19], therefore, when the atrium contracts, pulmonary vein ostia also constrict, thereby reducing their dimensions. The positive systolic RPA deflection begins just after the QRS (at the same instant as achieving the minimal diameter), consistent with blood entering the pulmonary artery from the right ventricle and distending it. The RPA then reaches its maximal diameter during the T wave, and then the deflection declines throughout diastole, reaching its nadir at the end of the QRS complex.

The echocardiographically derived RPV/RPA in healthy horses is approximately half that observed in healthy dogs, for both RPV_min_/RPA_min_ (0.51 vs 1.15) and RPV_max_/RPA_max_ (0.60 vs 1.07), but similar to that observed in healthy cats (0.51 vs 0.51 and 0.60 vs 0.67, respectively). Moreover, computed tomographic studies in humans reported an upper limit of the RPA diameter around 19.8 mm [20] while the diameters of pulmonary vein ostia ranged from 9 mm to 12.4 mm [21], which would result in RPV/RPA of approximately 0.45–0.62. These findings suggest that humans, cats and horses have similar RPV/RPA.

By comparing the RPV and RPA dimensions to the aortic dimension, one can determine if the small RPV/RPA is due to a larger RPA or a smaller RPV (or both). Horses and cats appear to have a relatively smaller RPV/Ao, and a relatively larger RPA_min_/Ao compared to dogs. However, horses had a smaller RPA_max_/Ao than dogs or cats. The fractional dimensional change of the RPA and RPV, calculated in our horses, had a median ΔRPA of 22%, similar to the value reported in cats (23%) [8], but lower than what has been reported in dogs (37% and 43%) [6,9]. Moreover, equine ΔRPA values were closer to the human value (28%) than the value reported for dogs. Pulmonary artery fractional dimensional change is inversely related to pulmonary arterial pressure in dogs [9,10] and humans [22,23]. Heathy horses have higher pulmonary arterial pressures [24] than healthy dogs [25] and humans, therefore we speculated that they could have decreased fractional dimensional change of the right pulmonary artery compared to other species. On the other hand, we found a median ΔRPV of 33%, lower than that obtained in dogs (37%) [6], cats (40%) [8] and humans (39% for right superior pulmonary vein and 31% for right inferior pulmonary vein in a MRI-derived study) [26].

We did not find any relationships of any of the indexed RPV or RPA variables with either BW or HR. This is not surprising, as indexing removes the effect of size, and allows for a single threshold value to be established for healthy horses.

We have measured the aortic diameter from the right parasternal short-axis view at aortic valve level, where the valve cusps were visible at the onset of the diastole, as previously described for dogs and cats [6,8]. Commonly, aortic diameter is measured from the right parasternal long axis (left ventricular outflow tract) view at the end of diastole in horses [12–14]. We preferred to use the view described in dogs and cats to compare the results obtained in the three different species. Indeed, aortic measurements differ significantly throughout diastole and are not clinically interchangeably in dogs [27]. Whether this occurs also in horses remains unknown. However, clinicians measuring RPV and RPA variables and indexing them to the aorta should note that aortic measurements in different views and different phases of the cardiac cycle might yield different results.

We did not include any small ponies or miniature horses in our study–therefore, whether these values apply to equids different from our study population remains to be determined. Furthermore, we did not evaluate any horses with cardiac pathology to determine whether any of the variables will prove useful in assessing cardiac status.

## Conclusions

Our study supports the feasibility of measuring RPV and RPA diameters using transthoracic M-mode echocardiography and provides reference intervals for various pulmonary vascular variables in healthy horses. Further studies of these variables in horses with cardiac diseases, are needed to determine the clinical applicability and utility.

## Supporting information

S1 File Raw data and linear regression analysis from the study. Clinical and echocardiographic variables in 70 healthy horses. Linear regression analysis to examine the effects of bodyweight and heart rate on the RPV and RPA variables.
(XLSX)
